# Tissue-Specific Expression of Transgenic Secreted ACE in Vasculature Can Restore Normal Kidney Functions, but Not Blood Pressure, of Ace-/- Mice

**DOI:** 10.1371/journal.pone.0087484

**Published:** 2014-01-27

**Authors:** Saurabh Chattopadhyay, Sean P. Kessler, Juliana Almada Colucci, Michifumi Yamashita, Preenie deS Senanayake, Ganes C. Sen

**Affiliations:** 1 Department of Molecular Genetics, Lerner Research Institute, Cleveland Clinic, Cleveland, Ohio, United States of America; 2 Department of Ophthalmic Research, Cole Eye Institute, Cleveland Clinic, Cleveland, Ohio, United States of America; Max-Delbrück Center for Molecular Medicine (MDC), Germany

## Abstract

Angiotensin-converting enzyme (ACE) regulates normal blood pressure and fluid homeostasis through its action in the renin-angiotensin-system (RAS). Ace-/- mice are smaller in size, have low blood pressure and defective kidney structure and functions. All of these defects are cured by transgenic expression of somatic ACE (sACE) in vascular endothelial cells of Ace-/- mice. sACE is expressed on the surface of vascular endothelial cells and undergoes a natural cleavage secretion process to generate a soluble form in the body fluids. Both the tissue-bound and the soluble forms of ACE are enzymatically active, and generate the vasoactive octapeptide Angiotensin II (Ang II) with equal efficiency. To assess the relative physiological roles of the secreted and the cell-bound forms of ACE, we expressed, in the vascular endothelial cells of Ace-/- mice, the ectodomain of sACE, which corresponded to only the secreted form of ACE. Our results demonstrated that the secreted form of ACE could normalize kidney functions and RAS integrity, growth and development of Ace-/- mice, but not their blood pressure. This study clearly demonstrates that the secreted form of ACE cannot replace the tissue-bound ACE for maintaining normal blood pressure; a suitable balance between the tissue-bound and the soluble forms of ACE is essential for maintaining all physiological functions of ACE.

## Introduction

The Renin-Angiotensin System (RAS) is a coordinated hormonal cascade that modulates fluid and electrolyte balance as well as blood pressure regulation [1]. In the classical pathway of the RAS, renin, which is secreted in the kidney juxtaglomerular apparatus in response to a wide variety of stimuli, acts on the precursor Angiotensinogen to generate the decapeptide Angiotensin I (Ang I) [2]. Angiotensin-converting enzyme (ACE) plays a central role in the RAS through generation of the octapeptide angiotensin II (Ang II) from its inactive precursor Ang I [3], [4]. Ang II induces vasoconstriction, aldosterone release, and other physiologic actions to raise blood pressure [5]. ACE inhibitors block the formation of Ang II and have been used to treat hypertension. ACE exists as two isoforms *viz*, somatic ACE (sACE), the larger isoform which is expressed mainly in the somatic tissues and the smaller isoform, germinal ACE (gACE), which is expressed in sperm cells. Both isoforms are synthesized from a single gene using tissue specific promoters and have distinct and tissue-specific functions in the body [6]–[8].

Studies with ACE knockout mice revealed additional roles of ACE beyond blood pressure regulation, especially in kidney functions, development and male fertility [9]–[11]. In addition to these functions, ACE plays roles in fat metabolism, inflammation and immune responses [12]. Our previous studies using transgenic ACE expression in Ace-/- mice demonstrated tissue- and isoform-specific physiological functions of ACE. The Ts mice, which express transgenic sACE only in the vascular endothelial cells of Ace-/- mice, can restore normal blood pressure [8]. However, gACE is not able to substitute for sACE for the maintenance of normal blood pressure of Ace-/- mice. Expression of gACE, but not sACE, in sperm successfully restores the male fertility of Ace-/- mice [7].

ACE is expressed on the cell surface as type I ectoprotein, with a long ectodomain containing the enzymatic active site, short cytoplasmic domain and a transmembrane domain [13]–[15]. The ectodomain of ACE can be cleaved by a membrane-bound metalloprotease, to generate a soluble form [16]–[18]. Although ACE secretase is unknown, its activity has been characterized as a membrane-associated protease. Previous studies have demonstrated that the cytoplasmic domain of ACE interacts with various signaling proteins such as, Calmodulin, Protein Kinase C, Casein Kinase 2 (CK2) and regulate the cleavage-secretion of ACE [19]–[22]. Moreover, the association of these proteins regulates the phosphorylation of ACE on a specific Ser residue in the cytoplasmic domain [19], [22]. Because both the cell-bound and the secreted forms of ACE are enzymatically active, their functional distinction has been of great interest. Cell-bound ACE, in addition to its enzymatic activity, can act as a signaling protein on the cell surface. Signaling via ACE activates CK2, which phosphorylates the cytoplasmic domain of ACE, suggesting phosphorylation of the cytoplasmic domain of ACE as a potential intracellular event which may contribute to the functions of ACE in the endothelial cells [23].

To investigate the relative roles of secreted and cell-bound ACE in various physiological functions of ACE, we have generated a transgenic mouse model which expressed only the secreted form of sACE, without any cell-bound form. This mutant ACE protein lacks the transmembrane and the cytoplasmic domains and, therefore, is constitutively secreted in the circulation. Our results demonstrated that the secreted ACE could not restore normal blood pressure of Ace-/- mice, although their kidney functions were normal. This study demonstrates that a cell-bound ACE is critical for maintaining normal blood pressure; a secreted ACE cannot substitute for the cell-bound form for this function.

## Materials and Methods

### Generation of target vector

We used Wt rabbit sACE cDNA to generate esACE, by deletion of the transmembrane and cytoplasmic domain coding region. The esACE cDNA was then used to generate Tie-esACE construct, as described previously [8]. The expression and secretion of ACE was tested by transient transfection of the tie-esACE plasmid in human fibrosarcoma cells (HT1080) followed by Western Blotting of the cell extracts and culture medium. The Tie-esACE-BGHpA transgene was subsequently released from this plasmid by SpeI and AsnI digestion and sent to the University of Cincinnati Transgenic Mouse Core Facility for the generation of transgenic mice utilizing standard techniques.

### Establishment of transgenic mice

The tie-esACE transgene was microinjected into the pronuclei of FVB strain zygotes using standard techniques. Adult FVB tie-esACE-BGHpA (TeS) transgenic founder mice (Ace+/+, TeS+/−) were mated with Ace+/− FVB mice to generate Ace+/− TeS+/− mice. Interbreeding between male and female Ace+/−, TeS+/− mice within the same line was performed to generate the Ace-/-, TeS/TeS (TeS). Genotyping of all mice was performed by Southern Blotting as described below. Expression of esACE in the transgenic line was confirmed by Western Blotting of the serum using anti-ACE antibody. All of the mice described in this study, including Wt, KO, Tie-esACE (TeS) and Tie-sACE (Ts), were of the FVB strain (Table 1). All mouse experimental protocols were approved by the Lerner Research Institute's Institutional Animal Care and Use Committee.

**Table 1:** Transgenic mice strains.

Strain	Transgene	Genotype	ACE isoform	Expression
**Wt**	None	Ace +/+	Somatic	Vascular endothelial cells
				Proximal tubule cells
				Brain
				Leydig cells
			Germinal	Sperm
**KO**	None	Ace -/-	None	None
**Ts**	Tie-sACE	Ace -/-Ts/Ts	Somatic	Vascular endothelial cells
**TeS**	Tie-esACE	Ace -/- TeS/TeS	Somatic	Vascular endothelial cells (sACE expressed only in serum)

### Maintenance of control mice

Ace-/- Ts/Ts (Ts) control mice were generated by mating Ace+/− Ts/Ts males with Ace-/- Ts/Ts females as described previously [8]. Ace-/- and Ace+/− control mice were generated by mating Ace+/− male mice with Ace+/− female mice. All of the mice described in this study, including Wt, KO, Tie-esACE (TeS) and Tie-sACE (Ts), were of the FVB strain (Table 1).

### Southern Blot hybridization

Southern blot genotyping was performed as described previously utilizing Sac I digestion of genomic tail snip DNA [8]. Heterozygosis or homozygosis of the transgene was determined by normalizing the transgene value to the endogenous mouse *Ace* gene value in the same genomic DNA sample using Imagequant software. The endogenous *Ace* genotype is determined by the presence of a wild-type 6.6 kB SacI genomic fragment or the disrupted 8.4 kB SacI (Ace-/-) genomic fragment [8]. The presence of the tie-esACE transgene is indicated by hybridization of the probe with a 3.7 kB fragment.

### ACE enzyme activity assay

The enzymatic activity of ACE was assayed using Hip-His-Leu as a substrate and measuring fluorimetrically the His-Leu liberated at 5 mM of Hip-His-Leu. Serum (1 µl) from retro-orbital eye bleed or tissue homogenates of the transgenic mice were used to measure ACE enzymatic activity. Activity values are reported as µmoles His-Leu per per µl of serum or 25 µg of tissue protein extract liberated from Hip-His-Leu after one-hour incubation at 37°C.

### Plasma renin activity

Blood was collected by retro-orbital sinus plexus eye bleed under brief isoflurane anesthesia and plasma renin activity was measured as described before [24]. Briefly, plasma renin activity, defined as the rate of Ang I generation from renin in the sample and excess exogenous substrate provided from nephrectomized rat plasma, was incubated at pH 6.5 (rats) and pH 8.5 (mice) for 90 minutes. Ang I generated in the sample was quantified by radioimmunoassay (DiaSorin Corp, Stillwater, MN).

### Serum creatinine measurement

Serum creatinine levels were determined by previously described alkaline picrate method [25]. Standards (Sigma-Aldrich) or 20 µl of serum were added into a 96-well microtiter plate. Alkaline picrate solution (10.8 mM picric acid, 29 mM sodium borate, 167 mM NaOH, 1.67% SDS) was added and incubated for 10 min at RT, absorbance was read at 490 nm. After the absorbance measurement, 60% acetic acid was added into all wells and left for 8 min at RT. The absorbance was read at 490 nm again, and subtracted from the first absorbance.

### Angiotensin II measurements

For each genotype, blood from four age matched adult mice was pooled to achieve a 1 ml plasma sample. Samples were concentrated on C18 columns (Sep-Pak columns; Waters), evaporated to dryness, and reconstituted in 0.9% NaCl, 0.03% acetic acid and 0.1% BSA). The levels of Ang II were quantified by RIA [26], [27]. The results represent the arithmetic mean of assaying a minimum of 5 pools from each genotype ±95% CI for the mean. Renal Ang II was measured from age- and genotype-matched mice, as described previously [28].

### Measurement of kidney functions

Age-matched adult mice (Wt, KO, Ts, TeS) were individually placed in a Nalgene metabolic cage supplied with powdered standard chow and water *ad libitum*. The data indicates average daily (24 h) water consumption and urine volume produced for five consecutive days for each of five mice of the same genotype. Urine osmolality was measured for each mouse using the Osmette A (Precision Instruments, Inc., Natick, MA) freezing point osmometer according to the manufacturer's instructions. Triplicate readings were performed on the urine collected as indicated above.

### Blood pressure measurement

Blood pressure was measured by two independent methods, by radiotelemetry and tail-cuff plethysmography. For telemetric measurements, the mice were anesthetized with isoflurane (3% in an oxygen stream). The BP transmitter (TA11PA-C10, Data Sciences International, St Paul, MN, USA) was implanted as follows: the catheter was inserted into the left common carotid artery, and the transmitter was positioned in the right flank [29]. The mice received 0.5 mL of 0.25% bupivicaine subcutaneously five minutes before surgery. After a 7 days recovery period each individual mouse cage was placed on the top of a radio-receiver (Model RPC-1) for measurement of BP, heart rate (HR), and spontaneous locomotor activity (SLA). Experimental data was recorded daily during 60 minutes for six consecutive days and analyzed using the Dataquest ART system, version 4.2 (Data Sciences International). A non-invasive computerized RTBP007 tail cuff blood pressure system (Harvard Apparatus, Holliston, MA) was used to obtain systolic blood pressure on conscious mice as described previously [27].

### Histology and immunohistochemistry

Kidneys from age-matched adult mice were paraffin embedded, cross-sectioned at 2.5 µm thickness and hematoxylin and eosin stained by the Histology Core (Lerner Research Institute, Cleveland, OH). Immunohistochemistry was performed following de-paraffinization as described previously [30]. Slides were incubated in 10 mM sodium citrate pH 6.0 for 30 min at 25°C, and then returned to PBS. The slides were blocked in PBS + 10% horse serum + 0.3% Triton X-100 (blocking buffer) for 2 h at 25°C. The polyclonal goat anti-ACE antibody, diluted 1∶1000 in blocking buffer was applied to a slide in a humid chamber for 16 h at 4°C. Following washes in PBS + 0.3% Triton X-100 (PBST), anti-goat-FITC (Santa Cruz) was applied to polyclonal ACE stained slides at 1∶3000 dilution in blocking buffer to each section for 2 h in the dark at 25°C. Following washes in PBST, Vectashield +/− DAPI (Vector Laboratories) diluted 1∶1 in PBS was applied. For Ang II staining, kidney slides were incubated with polyclonal rabbit anti-Ang II antibody (generated at Hybridoma Core, Cleveland Clinic, 1∶300), using the same protocol as described above. Following the PBS wash, color reaction was carried out using the EnVision + System-HRP (DAB) (Dako) according to the manufacturer's instructions. All stained slides were visualized with a Leica digital fluorescent microscope and processed in Adobe Photoshop software.

### Kidney-associated renin

Kidneys from Ace+/− or TeS mice were homogenized in 20 mM Tris HCl, 150 mM NaCl, 0.1% Triton X-100, protease inhibitors and phosphatase inhibitors leaving on ice for 30 min and spun at 14000 rpm at 4°C. The homogenates (50 µg total protein) were separated on 12% SDS-PAGE and Western blotted by anti-renin antibody (1∶1000, Abcam). The loading control was confirmed by Western blotting against actin.

### Statistics

Data are presented as arithmetic means and variations as 95% confidence interval of the mean. Significance values were obtained by unpaired *t* test, comparing experimental mice to the Wt mice or the KO mice as indicated in the figure legend. The statistical significance has been indicated in the figure legends.

## Results

### Generation of experimental mice

**Figure 1:**
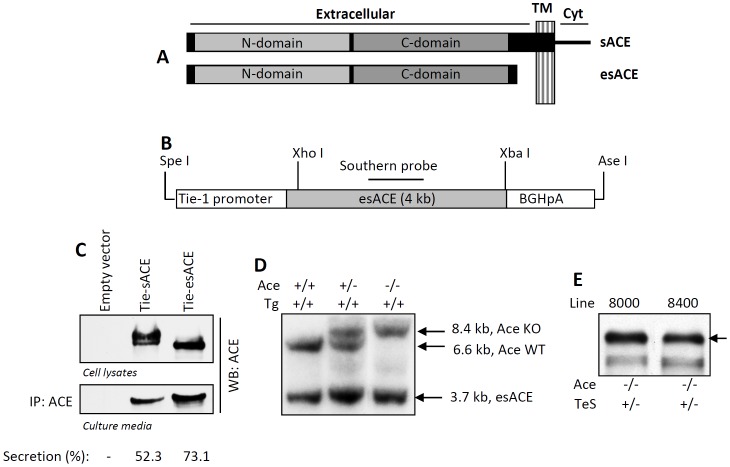
Generation of experimental mice. ***A*** Schematic representation of sACE and esACE is shown, different domains are indicated; TM, transmembrane domain, Cyt, cytoplasmic domain. ***B*** The Tie-1-esACE (rabbit somatic ACE without the transmembrane domain, as shown above)-BGHpA transgene (TeS) and rabbit ACE cDNA Southern Probe were designed. ***C*** The target vector (Tie-esACE) and the WT sACE (Tie-sACE) were transfected in HT1080 cells, the cell lysates and culture medium were analyzed for ACE by Western Blot by anti-ACE antibody. Secretion of ACE was measured as percentage of total ACE secreted into the culture media, by densitometric analyses. ***D*** A representative Southern Blot of the transgenic mice containing the Tie-esACE transgene (Tg), using the probe indicated in B, is shown. The genomic DNA, digested with Sac I, yielded a 3.7 kB transgene, a 6.6 kB Ace allele and an 8.4 kB disrupted Ace allele. ***E*** Serum obtained from retro-orbital eye bleed from TeS transgenic mice lines (8000 and 8400) were analyzed for ACE expression by Western Blot using anti-ACE antibody.

We have shown previously that the expression of Wt sACE in the vascular endothelial cells, which generates both cell-bound and secreted ACE, can restore normal blood pressure of Ace-/- mice [8]. In the present study, we investigated whether a secreted form of sACE could substitute for cell-bund ACE. We have designed a mutant somatic ACE which was missing the transmembrane and the cytoplasmic domains (esACE) (Figure 1A), and expressed in the vascular endothelial cells of Ace-/- mice. The tissue-specific Tie-esACE targeting vector was constructed from the parental Tie-sACE plasmid, by deletion of the transmembrane and the cytoplasmic domains of sACE (Figure 1B). The Tie-esACE-BGHpA transgene (TeS) was assembled as shown in Fig. 1B and tested *in vitro* by transiently expressing in human cells (HT1080). The esACE protein was expressed in these cells with similar expression as the sACE, with the expected size. This cellular form of esACE is the underglycosylated precursor form of ACE which is not transported to the cell surface; as indicated by two forms (the precursor and the glycosylated cell surface expressing form) for the sACE (Figure 1C, upper panel). As expected, the esACE protein was secreted relatively more to the culture media as compared to the sACE (cleavage-secretion of esACE: 73.1% vs. sACE: 52.3%, Figure 1C, lower panel). The esACE protein was enzymatically active, as tested by ACE activity assay using sACE as a control (data not shown). The SpeI-AseI fragment containing the TeS transgene (Figure 1B) was microinjected into the pronuclei of FVB zygotes and then implanted in the uteri of pseudopregnant mothers. The Tie-esACE (TeS) experimental mice were generated by crossing Ace+/− mice with mice carrying the TeS transgene. Two independent founder lines (8000 and 8400) carrying the TeS transgene were generated and these were separately crossed with Ace+/− mice and the progenies were genotyped by Southern Blot analysis for the presence of the transgene. Both transgenic mice lines transmitted the transgene to their progeny (Figure 1D). The transgenic lines were mated (Ace+/− TeS/TeS males with Ace+/− TeS/TeS females of the same line) to generate the Ace-/- TeS/TeS experimental mice. Serum obtained from retro-orbital eye bleed was used for testing secreted ACE protein; both transgenic TeS lines expressed comparable levels of esACE protein, detected by Western blot or enzymatic activity (Figure 1E and data not shown). Line 8400 was used for all the physiological analyses described in this study and the 8000 line was discontinued.

### Physiological properties of the experimental TeS mice

#### Abnormal fertility

The TeS mice exhibited normal growth rates, development and restored body weight and size, similar to Wt or Ts mice (data not shown) [8]. This indicates that the secreted sACE can substitute for the cell-bound sACE for maintaining these functions in mice. A striking phenotype of Ace-/- mice is male sterility; only a sperm specific expression of gACE restores the fertility of Ace-/- mice [31]. As expected, male fertility was not corrected in the TeS mice. Moreover, the presence of high levels of secreted ACE in the serum did not adversely affect or reduce the fertility of Ace+/+ or Ace+/− male mice.

#### Normal kidney structures and functions

**Figure 2:**
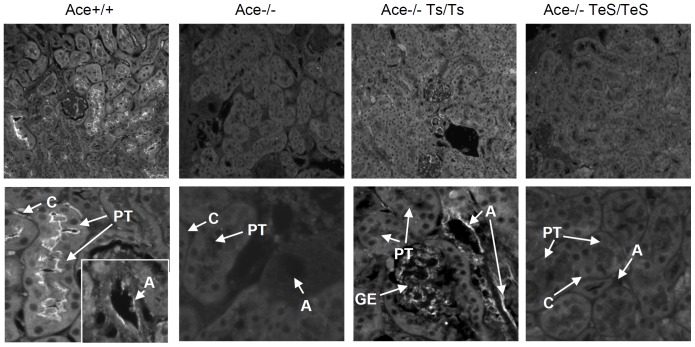
Experimental TeS mice do not express cell-bound ACE in the kidney. Age-matched adult kidneys from Wt (Ace+/+), ACE knockout (Ace-/-), two transgenic (Ace-/- Ts/Ts or Ace-/- TeS/TeS) mice were prepared and stained for ACE expression with anti-ACE polyclonal serum, followed by Alexa 568-conjugated secondary antibody (described in Materials and Methods). Slides were viewed with a Leica fluorescent microscope at 20X magnification. PT: proximal tubular epithelial cells, A: arterial endothelial cells, V: peritubular capillaries, GE: glomerular endothelial cells.

Since esACE was exclusively secreted into the blood flow from vascular endothelial cells, no significant ACE staining was observed in the kidney section of TeS mice by immunohistochemistry (Figure 2). The Ace+/+ mice showed positive ACE staining in the brush border membrane of the proximal tubular epithelial cells (PT), arterial endothelial cells (A), and peritubular capillaries (C). As expected, Ace-/- kidney did not show any ACE staining. The Ts mice showed positive ACE staining in arterial endothelial cells (A), glomerular endothelial cells (GE), but not in the proximal tubular (PT) cells. No ACE staining was detected in any area of the kidney section tested from TeS mice (Figure 2); confirming the absence of any cell-bound ACE in TeS mice.

**Figure 3:**
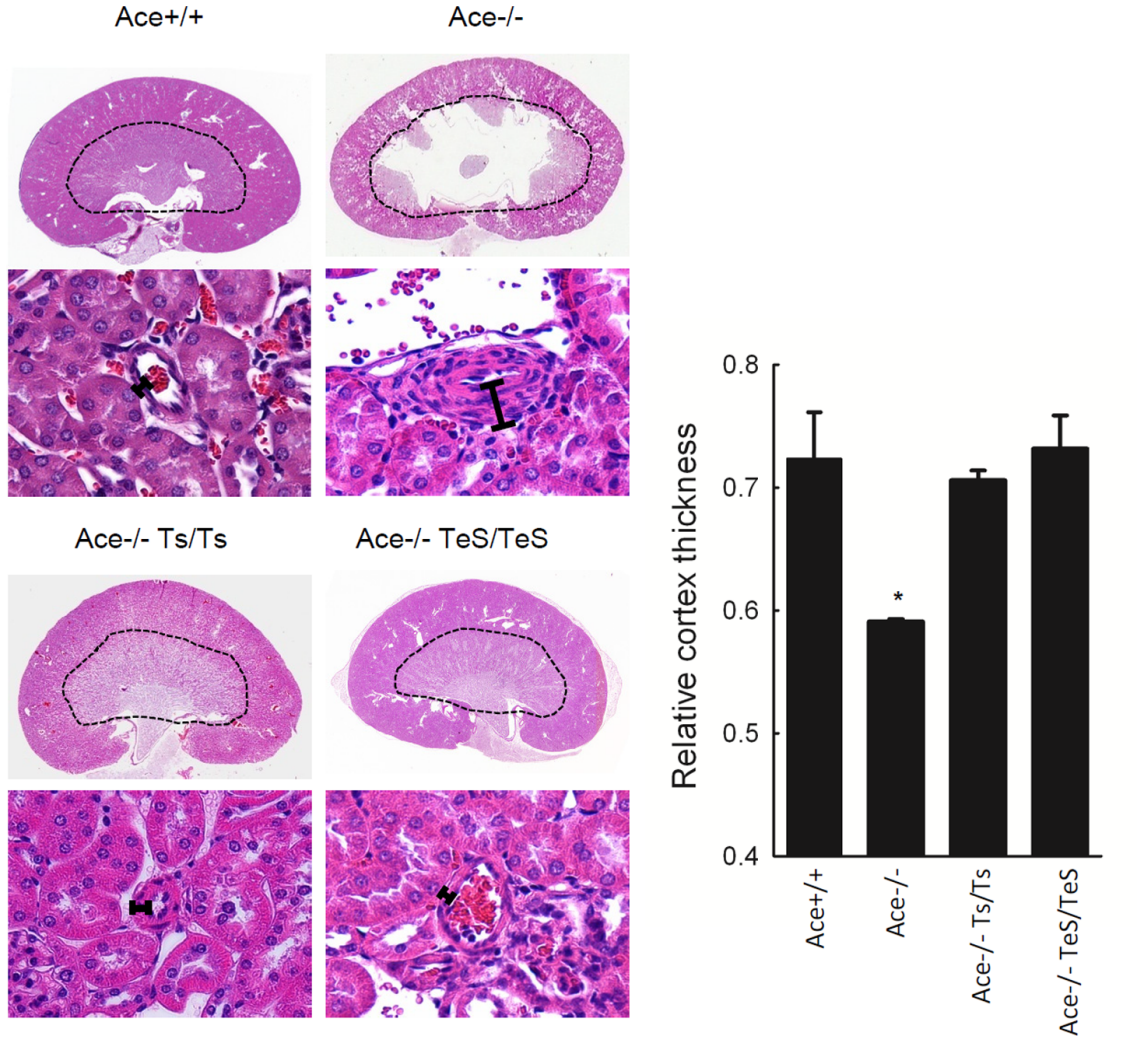
Secreted sACE expressing mice exhibit normal kidney structures. Hematoxylin and eosin-stained tissues from kidneys of age-matched adult mice are shown. All kidneys were photographed at 0.6X (whole cross section) or 20X (tubuli and arteriole) with a digital Leica light microscope. Manually drawn dotted lines indicate the borderline between the cortex and the medulla. The black bars represent wall thickness of arteriole. The lower panels show the vascular structures of the kidney sections. The relative cortex thickness was measured as a ratio between the medulla and the whole kidney section area. Values displayed as mean ± SD (* p<0.01).

Ace-/- mice exhibit hypoplasia of renal cortex; in the renal cross section, the area outside the dotted line is markedly reduced compared to the Ace+/+ mice (Figure 3, left panel). The development of the renal medulla of Ace-/- mice is also impaired: a large renal pelvis appears inside the dotted line in the center because the medulla is largely defective. The gross anatomy of the kidney from TeS mice was indistinguishable from that of Ace+/+ or the Ts mice, and the vessel wall thickness returned to normal (Figure 3, left panel). This was further confirmed by a quantitative analysis of relative cortex thickness from multiple kidney sections of these mice. The results clearly indicate that the TeS mice exhibited normal cortex thickness, which is similar to the Wt or Ts mice (Figure 3, right panel).

**Figure 4:**
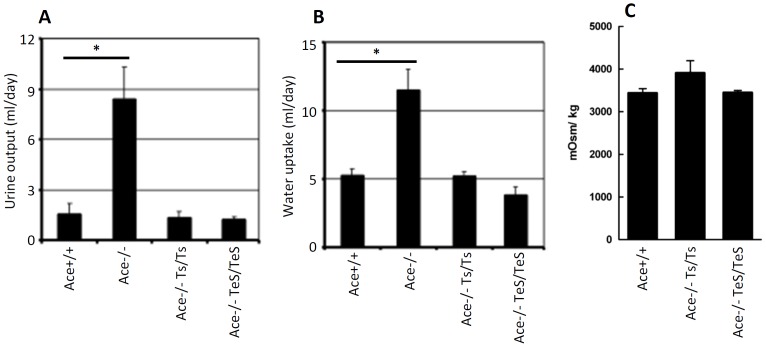
Secreted sACE expressing mice exhibit normal kidney functions. Five, age-matched adult mice (Wt, Ace+/+, KO, Ace-/-, Ts, Ace-/- Ts/Ts, TeS, Ace-/- TeS/TeS) were tested using metabolic cage for a period of five consecutive days. ***A*** Urine produced over a period of 24 h from WT, KO, Ts and TeS mice is shown. ***B*** Water uptake (in ml), during the same 24 h period. The data for the control mice (Wt, KO and Ts) were adopted from our previously published paper [8]. ***C*** Urine osmolality was measured for each mouse using the Osmette A (Precision Instruments, Inc., Natick, MA) freezing point osmometer. Values are displayed as mean ± SD (* *p*<0.001).

To address whether the restored kidney structures correspond to normal kidney functions, we compared their urine concentrating ability by measuring water intake, urine output and urine osmolarity. The overall fluid homeostasis was measured by the amount of urine output and water uptake over a period of five days. Consistent with previous findings, the Ace-/- mice exhibited significantly high urine output as well as high water uptake, presumably due to the defect in concentrating urine (Figure 4A, B). However, the average urine output and water uptake in the TeS mice were comparable to that observed in Wt or Ts mice (Figure 4A, B). In addition to this, we measured the urine osmolarity of the mice after overnight water restriction and the results indicate that the TeS mice showed comparable urine osmolarity to that of the Wt or Ts mice (Figure 4C). Therefore, circulating sACE could substitute for cell-bound sACE with respect to the restoration of normal kidney functions. These results, together with our previous studies using the Ts mice, further reinforces the notion that circulating sACE in serum is not only sufficient but can also substitute for the proximal tubular ACE, for maintaining normal kidney phenotypes.

#### Normal kidney-associated and circulating RAS activity

**Figure 5:**
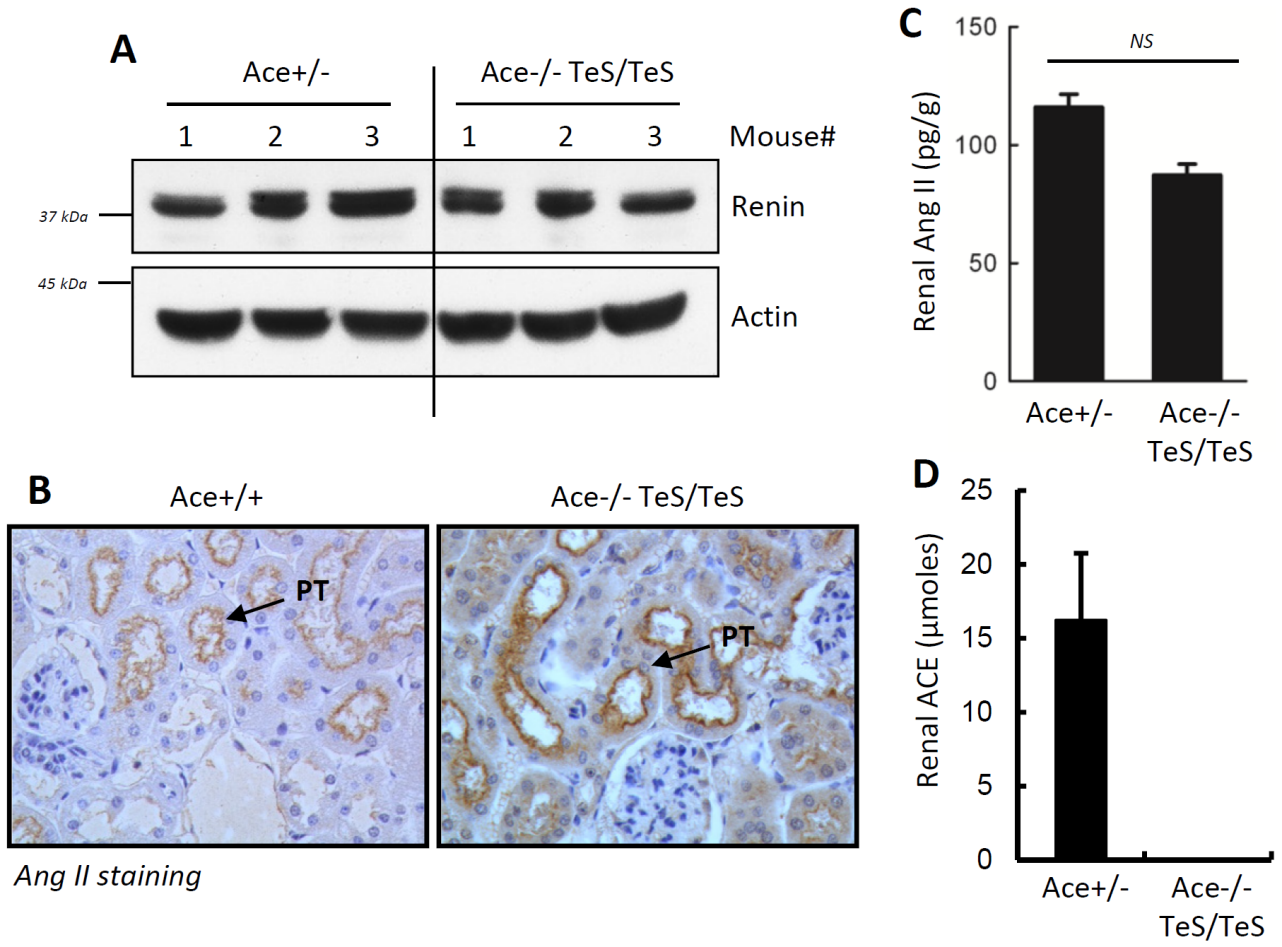
TeS mice exhibit normal kidney-associated RAS activity. ***A*** The kidney homogenates from Ace+/− and TeS mice were analyzed by SDS-PAGE followed by Western Blot against anti-renin antibody (Abcam). ***B*** Kidney slides from Wt and experimental TeS mice were stained with anti-AngII antibody followed by EnVision+System-HRP (DAB) detection. Slides were viewed as described in (A); PT: proximal tubular epithelial cells. ***C*** Renal Ang II levels were assayed as described in the Methods section. Ace +/− and Ace-/- TeS/TeS showed no significant difference in Ang II at the tissue level. ***D*** Age- and genotype-matched adult mice were used to measure ACE activity in kidney homogenates (per 25 µg of protein), by analyzing the liberated His-Leu using Hip-His-Leu as a substrate. Values are presented as mean values ± SD, from five mice.

**Figure 6:**
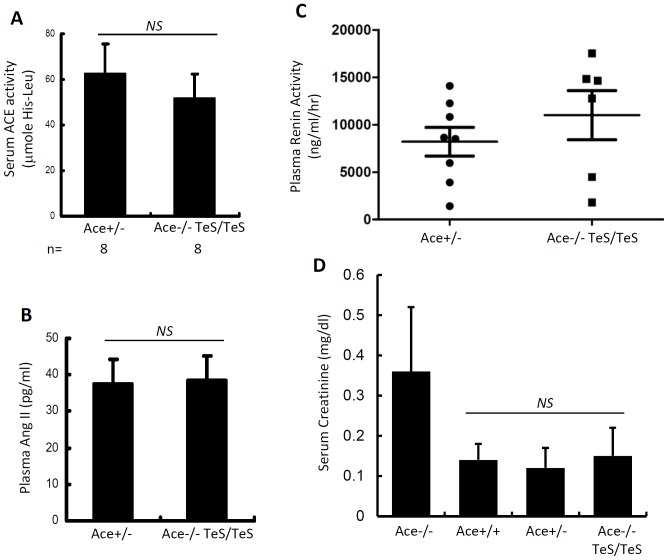
TeS mice exhibit normal circulating RAS activity. ***A*** Age- and genotype-matched adult mice were used to analyze ACE activity in the serum. The serum (1 µl) was assayed for ACE activity, measured as µmoles of His-Leu liberated from Hip-His-Leu in 1 h at 37°C. Each bar represents an average of multiple mice (number is indicated as n). ***B*** Age- and genotype-matched adult mice (three for each group) were used to pool plasma (1 ml) and assayed for Ang II levels (described in Materials and Methods). Each data point is an average of two independent measurements (*NS*, non-significant). ***C*** Age and genotype-matched adult mice (as indicated) were used to pool plasma and assayed for renin activity, as described in Materials and Methods. The difference in renin activity between the two groups of mice is not statistically significant (using paired t-test). ***D*** Serum collected from Ace-/-, Ace+/+, Ace+/− and TeS mice and used to measure creatinine levels. The difference between the three groups is not statistically significant.

We tested the integrity of local RAS by measuring kidney-associated renin and Ang II levels of TeS mice. Renin, a critical regulator of local RAS, was analyzed in kidney homogenates. The TeS mice expressed similar levels of kidney-associated renin as compared to Ace+/− mice (Figure 5A). To further investigate whether the normal renin expression correlates with Ang II level, we analyzed kidney-associated Ang II by immunostaining of kidney sections with anti-Ang II antibody. Comparable Ang II staining was observed in Ace+/+ and TeS proximal tubules, specifically in the brush border (Figure 5B). The immunostaining result was further confirmed by an independent assay (RIA) and by comparing with Ace+/− mice. The results indicate the TeS mice showed similar Ang II levels, compared to Ace+/− mice (Figure 5C). As expected, the TeS mice did not show any kidney-associated ACE activity, when compared with Ace+/− mice (Figure 5D). These results indicate that although cell-bound ACE was not present, TeS mice were not defective in kidney associated Ang II generation. To examine the circulating RAS activity, we analyzed ACE, Ang II and renin levels in the circulation. When compared to Ace+/− mice, the TeS mice expressed similar levels of soluble ACE (Figure 6A) and plasma Ang II (Figure 6B). The unaltered levels of plasma Ang II in the TeS mice was correlated with plasma renin activity, which was similar to the control Ace+/− mice (Figure 6C). No defects in kidney functions were observed for TeS mice even at a later stage of life, compared to the Ace-/- mice, which do not live longer. Serum creatinine level, a marker of kidney functions, was unaltered in TeS mice, when compared with Ace+/+ or Ace+/− control mice; however, the Ace-/- mice showed increased levels of serum creatinine (Figure 6D), indicating defective kidney functions. These results indicate that the absence of cell-bound ACE did not affect the RAS activity, in kidney or in circulation.

#### Low blood pressure

**Figure 7:**
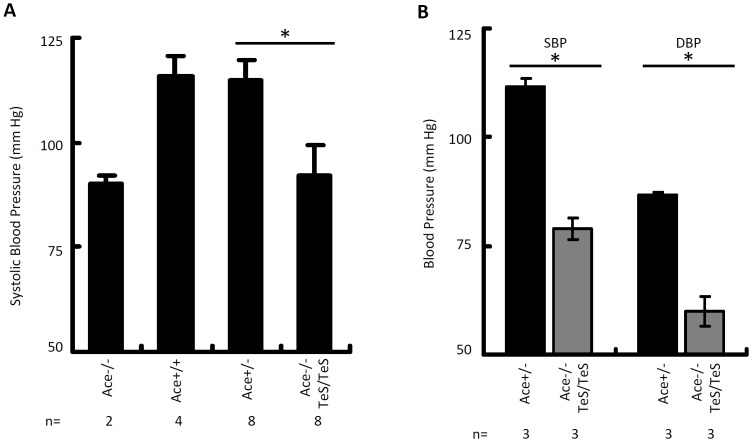
Secreted sACE expressing mice cannot maintain normal blood pressure. ***A*** The systolic blood pressure of age-matched adult mice (Ace-/-, Ace+/+, Ace+/− and TeS, as indicated) was measured using non-invasive, computerized tail-cuff plethysmography method. The systolic blood pressure (in mm Hg) was calculated using the mean daily blood pressure over a five day reading period (the number of mice is indicated as *n*; * *p*<0.001 Ace+/− *vs* Ace-/- TeS/TeS). ***B*** Ace+/− and Ace-/- TeS/TeS (TeS) mice were subjected to radiotelemetric analyses for measurement of both systolic (SBP and diastolic blood pressure (DBP) (described in Experimental Procedures) and a mean blood pressure is represented for each group of mice (n, the number of mice from each group; * *p*<0.001 Ace+/− *vs* Ace-/- TeS/TeS).

To examine whether restored renal phenotype, normal levels of circulating ACE, renal and plasma Ang II, plasma renin activity are sufficient to maintain the normal blood pressure of TeS mice, we conducted two independent experiments, computerized tail-cuff plethysmography and radiotelemetry, to measure the systolic and diastolic blood pressures of the mice. The TeS mice exhibited low systolic blood pressure, comparable to that of Ace-/- mice, whereas Wt (Ace+/+) or Ace+/− mice both showed normal blood pressure (Figure 7A). The systolic blood pressure of the TeS mice was 92.3±6.9 mm, compared to 90±2 mm of Ace-/- mice. In contrast, the corresponding numbers for Ace+/+ and Ace+/− mice were 116±4.7 mm and 115±4.6 mm respectively. Although we have successfully used tail-cuff method previously to determine the systolic blood pressure of conscious mice, we understand that this type of measurement impose substantial amounts of thermal and restrain stress that are known to affect blood pressure, heart rate and stress hormones [32]. To address this potential problem, we used wireless radio-telemetry as an additional assay. This technique has the advantage of allowing continuous, direct measurements of blood pressure without the need for restraints. Moreover, the telemetric analysis allowed us to measure diastolic blood pressure, in addition to systolic blood pressure, of the TeS mice. As shown in Figure 7B, the TeS mice exhibited lower systolic and diastolic blood pressures as compared to the Ace+/− mice. From the two independent measurements, we concluded that the TeS mice, which expressed similar levels of circulating ACE and plasma Ang II as the Ace+/− mice, could not maintain normal blood pressure. These results clearly indicate that the secreted form of sACE is not sufficient for maintaining normal blood pressure in mice.

## Discussion

Ectodomain shedding of ACE generates an enzymatically active secreted form, whose function is currently unknown. We hypothesized that a suitable balance between the cell-bound and secreted forms of ACE is critical for maintaining the normal physiological functions of the enzyme. To test this hypothesis, we generated transgenic mice expressing a secreted sACE, in the absence of any cell-bound ACE. Our results showed that secreted sACE could not maintain normal blood pressure; however, it was sufficient for restoring normal growth, development, kidney structures and functions, of Ace-/- mice. This strongly indicates the requirement of endothelial cell-bound sACE for maintaining normal blood pressure. This conclusion is supported by our previous results with another transgenic mouse, in which sACE was restrictedly expressed in the kidney proximal tubular cells (Gs mice) [8]. These mice expressed high levels of circulating sACE in the serum. Although the Gs mice exhibited normal kidney development and functions, the high level of circulating sACE in the serum was unable to restore normal blood pressure. In another study, a transgenic mouse expressing only the N-domain of sACE in the serum has been generated [33]. The circulating truncated sACE expressed in these mice cannot restore normal blood pressure; however, the observed defect could be due to the absence of the C-domain enzymatic site in the protein. In contrast, our study is based on a rational design of a transgenic mouse expressing the entire extracellular domain of sACE, which is the physiological soluble form of ACE in the circulation, by endothelial cells. The cleavage and secretion of ACE is negatively regulated by its cytoplasmic domain. *In vitro* studies using both sACE and gACE proteins show that deletion of cytoplasmic domain leads to elevated levels of soluble ACE [34], [35]. The pathological effects of ACE cleavage have been observed in patients with natural mutations of ACE. Studies indicate that mutations of sACE that generate cytoplasmic domain deleted ACE, lead to high levels of circulating ACE, which correlates to disorder in RAS activities [36], [37].

Inhibition of ACE enzymatic activity by selective ACE inhibitors blocks the generation of Ang II and thereby acts as a suitable approach for the treatment of high blood pressure. However, it remains unclear whether the Ang II generating function of ACE is the only link between this protein and the maintenance of normal blood pressure. Our study indicates that the level of Ang II is not an accurate indicator of ACE function in blood pressure maintenance. Although, both the TeS mice and the control Ace+/− mice expressed similar levels of renal and plasma Ang II (Figure 5C, 6B), they could not maintain normal blood pressure (Figure 7). Similar to the TeS mice, the Gs mice, which expressed high levels of plasma Ang II, was also unable to maintain normal blood pressure. Kidney associated RAS activity has also been shown to be involved in blood pressure regulation [38]. Surprisingly, TeS mice exhibited normal levels of kidney Ang II; however, this was not sufficient to restore the normal blood pressure.

So, why is cell-bound ACE essential for blood pressure maintenance? The concept of local vs. systemic RAS may be relevant to this question. The components of the RAS exist in many organs and are postulated to regulate tissue specific Ang II production [39]. Initially considered only a systemic process, the Ang II produced at the tissue level is now accounted for the regulation of the vasculature structure and tone in the microenvironment of each tissue [40]. These local systems depend critically on the presence of tissue-bound ACE and would be, therefore, presumably nonfunctional in the experimental TeS mice because secreted sACE, although capable of generating circulating Ang II in serum, is not able to generate local Ang II in the vascular endothelial bed. However, in addition to normal levels of serum Ang II, TeS mice exhibited kidney Ang II levels comparable to Wt mice. Although, it is not clear at this point, the kidney associated Ang II might be generated by ACE-independent alternative pathways. Multiple studies have described the growing importance of chymase-dependent pathways in generation of Ang II from Ang I [41]. Ang II, derived from ACE or chymase have also been suggested to have distinct physiological implications. The Ang II levels observed in the TeS mice suggest an alternative hypothesis, that sACE has dual functions, both of which are needed for blood pressure maintenance. One function depends on the traditional enzyme activity ACE, which is responsible for Ang II formation and bradykinin degradation. In the second function, ACE works as a receptor on the surface of endothelial cells and mediates outside-in signaling in them [42], [43]. There is strong evidence for the cytoplasmic domain of ACE being capable of activating intracellular signaling [23], a function of ACE that is independent of its enzymatic activity. The ligands that can trigger ACE signaling include ACE inhibitors, which bind to it strongly. Surprisingly, Ang II, a product of the enzyme activity of ACE, can also activate intracellular calcium signaling cascades [44]. Thus, the two alternative models for justifying the need of tissue-bound ACE, for blood pressure maintenance, may be interdependent. Tissue-bound ACE may be needed to produce high levels of Ang II locally, which, in turn, may trigger intracellular signaling by binding to cell-surface ACE. In future, one potential system for testing the relative contributions of the two models will be a genetically engineered mouse expressing, in its endothelial cells, cell-bound ACE that lacks the cytoplasmic tail. Such an ACE mutant will still produce ample quantities of local Ang II in the endothelium but will be incapable of producing intracellular signals. These mutant mice should have low blood pressure, if cytoplasmic-tail signaling is the critical determinant.
